# Propranolol inhibits EMT and metastasis in breast cancer through miR-499-5p-mediated Sox6

**DOI:** 10.1007/s00432-023-05599-w

**Published:** 2024-01-31

**Authors:** Bo Zheng, PeiXin Du, Zhi Zeng, Peng Cao, Xuelei Ma, Yu Jiang

**Affiliations:** 1 Health Management Center, Department of Oncology, West China Hospital, Sichuan University, Chengdu, 610041 China; 2 Institute for Breast Health Medicine, State Key Laboratory of Biotherapy, West China Hospital, Sichuan University, Chengdu, 610041 Sichuan China; 3 Huaxi Clinical College, Sichuan University, Chengdu, 610041 China; 4 Colorectal Cancer Center, West China Hospital, Sichuan University, Chengdu, 610041 China; 5 Department of Biotherapy, State Key Laboratory of Biotherapy and Cancer Center, West China Hospital, Sichuan University, and Collaborative Innovation Center of Biotherapy, Chengdu, 610041 China; 6 Department of Medical Oncology, Cancer Center, State Key Laboratory of Biotherapy, West China Hospital, Sichuan University, Sichuan, 610041 China

**Keywords:** Epithelial–mesenchymal transition, Propranolol, Breast cancer, 4T1 cells

## Abstract

**Purpose:**

This study will focus on 4T1 cells, a murine mammary adenocarcinoma cell line, as the primary research subject. We aim to investigate the inhibitory effects and mechanisms of propranolol on epithelial–mesenchymal transition (EMT) in breast cancer cells, aiming to elucidate this phenomenon at the miRNA level.

**Methods:**

In this study, the EMT inhibitory effect of propranolol was observed through in vitro and animal experiments. For the screening of potential target miRNAs and downstream target genes, second-generation sequencing (SGS) and bioinformatics analysis were conducted. Following the screening process, the identified target miRNAs and their respective target genes were confirmed using various experimental methods. To confirm the target miRNAs and target genes, Western Blot (WB), reverse transcription polymerase chain reaction (RT-PCR), and immunofluorescence experiments were performed.

**Results:**

In this study, we found that propranolol significantly reduced lung metastasis in 4T1 murine breast cancer cells (*p* < 0.05). In vitro and in vivo experiments demonstrated that propranolol inhibited the epithelial–mesenchymal transition (EMT) as evidenced by Western Blot analysis (*p* < 0.05). Through next-generation sequencing (SGS), subsequent bioinformatics analysis, and PCR validation, we identified a marked downregulation of miR-499-5p (*p* < 0.05), suggesting its potential involvement in mediating the suppressive effects of propranolol on EMT. Overexpression of miR-499-5p promoted EMT, migration, and invasion of 4T1 cells, and these effects were not reversed or attenuated by propranolol (Validated via Western Blot, wound healing assay, transwell migration, and invasion assays, *p* < 0.05). Sox6 was identified as a functional target of miR-499-5p, with its downregulation correlating with the observed EMT changes (*p* < 0.05). Silencing Sox6 or overexpressing miR-499-5p inhibited Sox6 expression, further promoting the processes of EMT, invasion, and migration in 4T1 cells. Notably, these effects were not alleviated by propranolol (validated via Western Blot, wound healing assay, transwell migration, and invasion assays, *p* < 0.05). The direct interaction between miR-499-5p and Sox6 mRNA was confirmed by dual-luciferase reporter gene assay.

**Conclusion:**

These results suggest that propranolol may have potential as a therapeutic agent for breast cancer treatment by targeting EMT and its regulatory mechanisms.

## Introduction

Breast cancer is the most commonly occurring tumor in women and remains one of the leading causes of cancer-related deaths worldwide (Siegel et al. 2020). Although the breast cancer mortality rate has gradually decreased due to advances in treatment modalities and the availability of screening (Smith et al. 2019), research on the successful treatment of breast cancer remains inconclusive. Propranolol (Prop) is a classical β-adrenergic receptor (β-AR) blocker that is widely used in the cardiovascular field (Friedman et al. 1986). Recently, studies have suggested that propranolol may have potential as a treatment option for tumors, as it has been shown to reduce the cumulative specific mortality (Barron et al. 2011) and recurrence rate in breast cancer patients (Choy et al. 2016). Breast cancer-related mortality is usually associated with the development of metastases in the brain, lungs, and bones (Fang et al. 2012; Langlands et al. 2013). Several studies have indicated that propranolol may reduce the incidence of metastasis in breast cancer patients (Choy et al. 2016; Powe et al. 2010) and may also reduce the migration and invasion of breast cancer cells in combination with metformin. Additionally, a particular study reported that propranolol is capable of reducing the incidence of lung metastasis in hormonal mice (Rico et al. 2017).

Epithelial–mesenchymal transition (EMT) plays a vital role in tumor metastasis (Kalluri and Weinberg 2009). It is generally believed that EMT drives the invasion and migration of various types of tumors, including breast cancer (Mittal 2018; Holland 2007). During EMT, there is typically a reduction in the expression of molecular markers associated with epithelial cells, such as E-cadherin, while the expression of mesenchymal markers like N-cadherin and vimentin is increased (Nijkamp et al. 2011). In our previous research, we demonstrated that propranolol antagonizes the promoting effect of norepinephrine on EMT in HT-29 and A549 cells (Zhang et al. 2016). Recent evidence also suggests that propranolol may have an inhibitory effect on EMT in breast cancer patients (Shaashua et al. 2017). However, the precise mechanisms underlying these effects are not yet fully understood.

MicroRNAs (miRNAs, miRs) are a class of non-coding RNAs that are approximately 18–25 nucleotides in length and can regulate gene expression at both the transcriptional and post-transcriptional levels (Bartel 2009). They play a critical role in various cellular processes, including proliferation, differentiation, apoptosis, invasion, and migration (Yu et al. 2010; Ma et al. 2017). In particular, microRNA-499-5p (miRNA-499-5p, miR-499-5p) has been implicated in the regulation of the EMT process in various tumors (He et al. 2019; Tauriello et al. 2010; Li et al. 2016). Furthermore, it has been demonstrated that Sox6 is a target gene of miRNA-499-5p (Li et al. 2013; Zhang et al. 2015; Wang et al. 2017), and recent studies have suggested that Sox6 may function as an EMT inhibitory factor in tumors, including breast cancer (Wang et al. 2018; Jiang et al. 2018).

In this study, we utilized second-generation sequencing (SGS) to explore the impact of propranolol on miRNA expression in breast cancer, specifically focusing on its effects on metastasis inhibition and EMT downregulation. Through in vitro experiments aimed at validating miRNAs displaying significant expression changes in the sequencing data and an extensive literature search for those miRNAs consistently exhibiting significant changes, we discovered a potential strong association of miR-499-5p with EMT. This finding was further confirmed in subsequent overexpression experiments. Subsequently, we cross-referenced the predicted target genes of miR-499-5p with the mRNA sequencing data, identifying mRNAs exhibiting consistent expression changes. Further validation of potential target genes influencing EMT was conducted through in vitro experiments following a comprehensive literature review. Ultimately, we hypothesized and confirmed that miR-499-5p might mediate the suppressive effects of propranolol on EMT and metastasis in breast cancer 4T1 cells by targeting Sox6. These findings provide important insights into potential therapeutic targets and offer a foundation for the development of novel breast cancer treatments.

## Results

### Propranolol inhibits lung metastasis and EMT in breast cancer in vivo

**Fig. 1:**
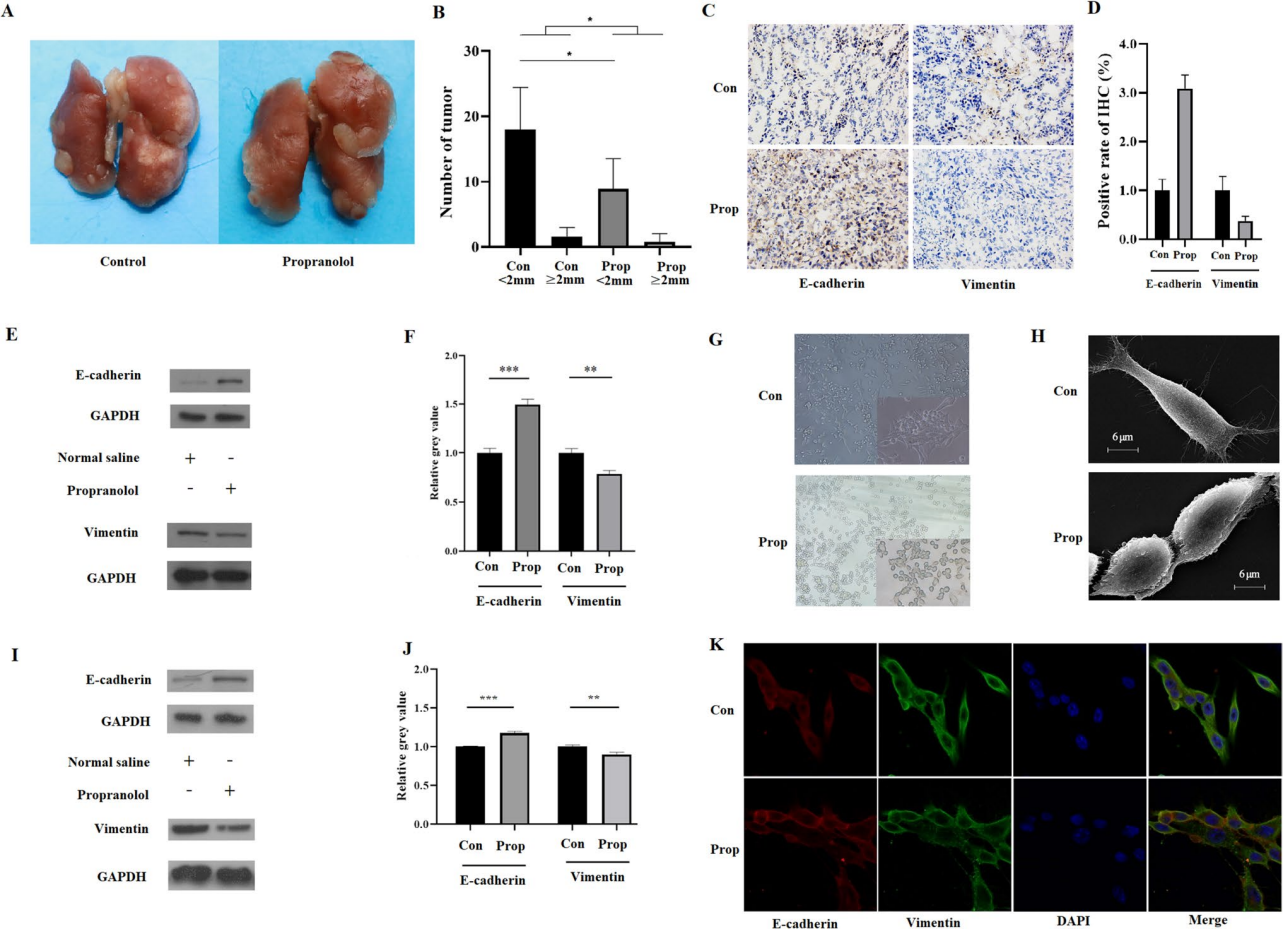
Propranolol inhibits lung metastases and EMT of 4T1 cells in vivo and in vitro. **A** Representative lung images from the control group and propranolol group mouse. **B** Comparison of the number of metastatic nodules with different diameters in lung tissue. The histogram shows the number of lung metastatic nodules in the control group and propranolol group. **C**, **D** Immunohistochemical staining of E-cadherin and vimentin protein in tumors from control and propranolol group. The fluorescence intensity was quantified and shown in histogram. **E**, **F** Western blot analysis of E-cadherin, vimentin, and GAPDH protein in tumors from control and propranolol group mouse. The histogram shows the intensity analysis of E-cadherin and vimentin normalized to GAPDH. **G**, **H** Light micrographs and electron micrograph of 4T1 cells treated with 10 μM propranolol or normal saline (NS) for 24 h, 40 × in light micrographs (lower right corner is 100 ×), 3000 × in electron micrograph. **I**, **J** Western blot analysis of E-cadherin, vimentin, and GAPDH protein in 4T1 cells treated with 10 μM propranolol or NS for 72 h. Histogram shows the intensity analysis of E-cadherin and vimentin normalized to GAPDH. **K** Immunofluorescence images of E-cadherin and vimentin in 4T1 cells treated with 10 μM propranolol or NS for 48 h. E-cadherin, vimentin, and cell nucleus are shown in red, green, and blue. *, *P* < 0.05; **, *P* < 0.01; ***, *P* < 0.001

To investigate the potential of propranolol to reduce tumor lung metastasis, we constructed a 4T1 tumor-bearing mouse model. After allowing tumors to form for 7 days, the mice were assigned to different treatment groups (see Methods for details). Following continuous treatment for 28 days, the mice were euthanized via cervical dislocation, and their lung tissue was collected for analysis of metastatic nodules. Our results demonstrated that propranolol treatment significantly reduced the number of lung metastatic nodules, both small (defined as diameter < 2 mm) and large (defined as diameter ≥ 2 mm) (Fig. 1A, B). Furthermore, immunohistochemistry and Western blot analyses indicated that propranolol inhibited EMT in vivo, as evidenced by the upregulation of E-cadherin and downregulation of vimentin in tumor tissue (implantation site) in the propranolol group compared to the control group (Fig. 1C–F).

### EMT inhibition by propranolol in breast cancer cells in vitro

To determine whether EMT inhibition by propranolol could be recapitulated in vitro, we cultured 4T1 cells and treated them with 10 μM propranolol. Previous studies have shown that 4T1 cells express a certain level of mesenchymal markers (Lou et al. 2008; Elisha et al. 2018; Li et al. 2017) and are considered to exhibit mesenchymal characteristics(Chou et al. 2014). Therefore, in line with prior studies, subsequent researchers employed 4T1 cells as a model system to evaluate the inhibitory effect of EMT (Li et al. 2017). Under light microscope, we observed that the control cells appeared more elongated and separated, with more pronounced mesenchymal features, than the propranolol-treated cells (Fig. 1G). Electron tunneling scanning microscopy confirmed that the control cells were longer and had more pseudopodia and microvilli than the propranolol-treated cells (Fig. 1H). Furthermore, Western blot and immunofluorescence analyses indicated that propranolol treatment increased the expression of E-cadherin and decreased the expression of vimentin in 4T1 cells consistent with EMT inhibition (Fig. 1I–K).

#### Propranolol’s inhibitory effects on metastasis and EMT are related to the downregulation of miRNA-499-5p

**Fig. 2:**
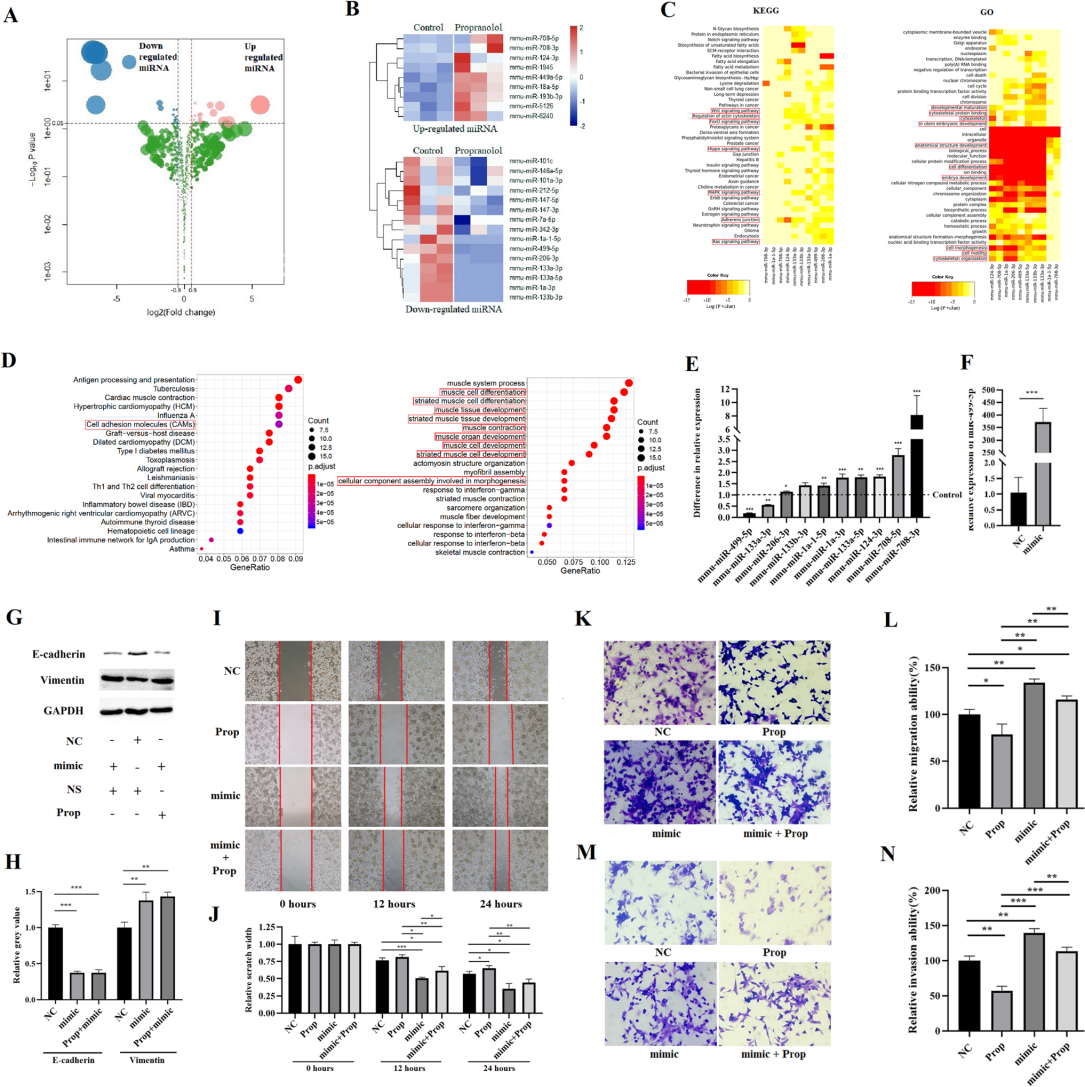
Inhibition of EMT in 4T1 cells by propranolol is mediated by miRNA-499-5p. **A** Volcano plot of differentially expressed miRNA between control and propranolol groups. **B** Heatmap of differentially expressed miRNA between control and propranolol groups (*P* < 0.05, two-tailed Student’s *t*-test). **C, D** Enrichment analysis of differentially expressed miRNA (**C**) and mRNA (**D**) between control and propranolol groups, with red boxes indicating EMT-related domains. **E** RT-PCR analysis of changes in miRNAs after treatment with 10 μM propranolol or NS for 48 h. The ΔΔCt method was used with U6 as an internal control for normalization. **F** The transfection efficiency of miR-499-5p mimic was detected using RT-PCR. **G, H** Western blot analysis of E-cadherin, vimentin, and GAPDH protein in 4T1 cells transfected with NC or miR-499-5p mimic and treated with 10 μM propranolol or NS for 72 h, with corresponding histogram of E-cadherin and vimentin intensity normalized to GAPDH. **I, J** Wound healing assay was used to measure the migration ability of 4T1 cells transfected with NC or miR-499-5p mimic and treated with 10 μM propranolol or NS, with corresponding histograms.** K–N** Transwell migration (**I**, **J**) and invasion (k, l) assays were used to measure the migration and invasion ability of 4T1 cells transfected with NC or miR-499-5p mimic and treated with 10 μM propranolol or NS, with corresponding histograms. *, *P* < 0.05; **, *P* < 0.01; ***, *P* < 0.001

To investigate the mechanism by which propranolol inhibits metastasis and EMT, we performed second-generation high-throughput sequencing of miRNA and mRNA expression in the tumor tissues of the propranolol group and control group. We then analyzed the sequencing data and found that compared to the control group, the propranolol group showed nine upregulated miRNAs and 15 downregulated miRNAs (*p* < 0.05) (Fig. 2A, B). Ten miRNAs with significant changes (|log2 (FoldChange)|> 2) were subjected to bioinformatics analysis (enrichment analysis) using the DIANA tool (http://diana.imis.athena-innovation.gr/), which is based on miRTarBase, microT-CDS, and TargetScan. Furthermore, three miRNA target gene prediction databases were used for enrichment analysis of the target gene prediction results of the abovementioned miRNAs, including Kyoto Encyclopedia of Genes and Genomes (KEGG) and Gene Ontology (GO). Our analysis revealed that the target genes of these miRNAs are closely associated with EMT-related domains, such as adhesion junctions, cell differentiation, and cytoskeleton (Fig. 2C). Enrichment analysis of mRNA sequencing results obtained similar results (Fig. 2D). These results suggest that propranolol may mediate the alteration of EMT-related protein expression through miRNAs, thus altering EMT at the cellular level.

Next, we treated 4T1 cells in vitro with propranolol and verified the changes in the ten miRNAs using reverse transcription polymerase chain reaction (RT-PCR). Our results indicated that miRNA-499-5p, miRNA-708-5p, and miRNA-708-3p changed significantly, and these findings were consistent with the sequencing results (2-△△Ct > 2 or < 0.5) (Fig. 2E). We selected miRNA-499-5p for further study as it has been reported to be associated with EMT in previous literature (He et al. 2019; Li et al. 2016; Long and Pi 2018; Liu et al. 2011), and there is strong evidence suggesting that miRNA-499-5p targets proteins closely related to EMT, such as Pten (Lin et al. 2010), Fzd8 (Murillo-Garzón et al. 2018), and Sox6.

To determine the effect of miRNA-499-5p on propranolol-regulated EMT, we transfected miRNA-499-5p mimics into 4T1 cells cultured in vitro to mimic overexpressed endogenous miRNA-499-5p, and RT-PCR was used to confirm transfection efficacy (Fig. 2F). Western blot analysis showed that the expression of E-cadherin was downregulated, while vimentin was upregulated in 4T1 cells transfected with miRNA-499-5p mimics when compared to those transfected with negative control mimics (NC), indicating that the cells underwent EMT. Moreover, this change could not be reversed or attenuated by propranolol (Fig. 2G, H). In addition, in the wound healing, cell migration, and invasion assay, we found that the migration and invasion rate of 4T1 cells transfected with miRNA-499-5p mimics were significantly increased compared to those transfected with negative control mimics, and propranolol could only slightly reduce this change (Fig. 2I–N). These results suggest that when miRNA-499-5p is overexpressed, propranolol's inhibitory effect on EMT and cell migration and invasion cannot be exerted. This indicates that the downregulation of miRNA-499-5p expression may be associated with propranolol’s inhibition of 4T1 cell migration, invasion, and EMT.

### miRNA-499-5p mediates the inhibitory effect of propranolol on EMT in 4T1 cells by targeting Sox6

**Fig. 3:**
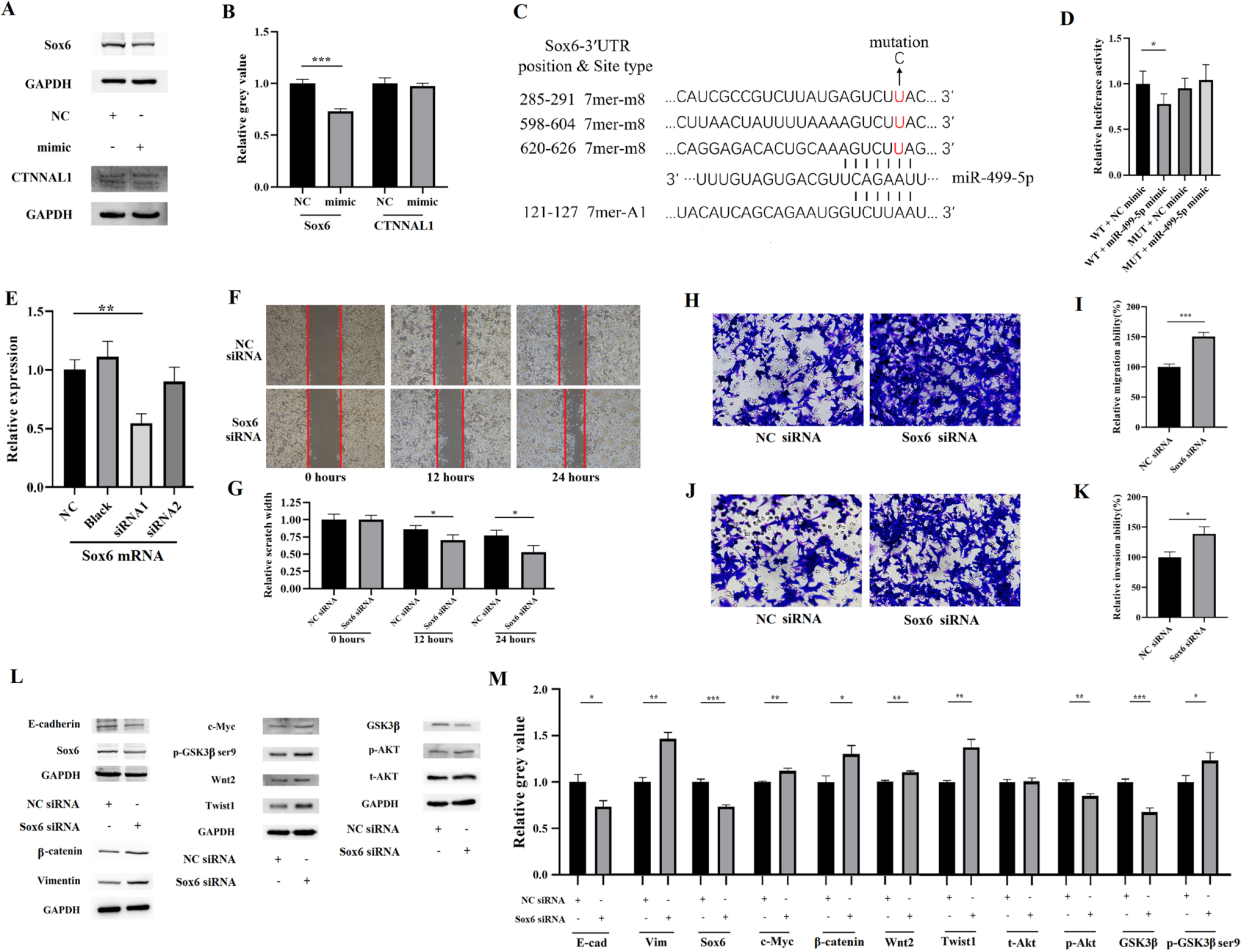
miR-499-5p regulates EMT in 4T1 cells via Sox6. **A**, **B** Western blot analysis of Sox6, CTNNAL1, and GAPDH protein in 4T1 cells transfected with NC or miR-499-5p mimic for 72 h, with corresponding histogram of Sox6 and CTNNAL1 normalized to GAPDH. **C** The binding sites of miR-499-5p to Sox6, of which three 7mer-m8 sites were used to construct the reporter plasmid, and the mutation sites are marked in red. **D** Histogram of the fluorescence intensity in the dual-luciferase reporter gene assay to confirm the targeting relationship between mi-499-5p and Sox6. **e** RT-PCR for detecting the knockdown efficiency of siRNA on Sox6 in 4T1 cells. **F**, **G** Wound healing assay was used to measure the migration ability of 4T1 cells transfected with Sox6 siRNA or NC siRNA, with corresponding histograms. **H–K** Transwell migration (**I**, **J**) and invasion (k, l) assays were used to measure the migration and invasion ability of 4T1 cells transfected with Sox6 siRNA or NC siRNA, with corresponding histograms. **L**, **M** Western blot analysis of Sox6, EMT-related and Sox6 downstream-related proteins, and GAPDH in 4T1 cells transfected with NC or Sox6 siRNA for 72 h, and corresponding histogram normalized to GAPDH.*, *P* < 0.05; **, *P* < 0.01; ***, *P* < 0.001

To determine the target of miRNA-499-5p during the EMT process, we used TargetScan and DIANA to predict the mRNAs targeted by miRNA-499-5p and compared these results with the mRNA sequencing results. The analysis indicated that 15 of the 464 upregulated mRNAs were consistent with 757 miRNA-499-5p target genes (Table 1). After reviewing the literature, we found that CTNNAL1 and Sox6 among these 15 mRNAs were closely associated with EMT (Jiang et al. 2018; Tan et al. 2018). Additionally, previous studies confirmed that Sox6 is a target of miRNA-499-5p (Li et al. 2013). Subsequent Western blot analysis demonstrated that the expression of Sox6 in 4T1 cells transfected with miRNA-499-5p was reduced, while the expression of CTNNAL1 did not change significantly (Fig. 3A, B). These results established Sox6 as a candidate target for further investigation. TargetScan predicted that miR-499-5p and Sox6 mRNA have similar binding sites (Fig. 3C). Furthermore, the results of the luciferase reporter gene assay indicated that in 4T1 cells, the fluorescence intensity was reduced in the group transfected with the Sox6-wild-type (WT) and miRNA-499-5p mimic compared to other groups, including those transfected with Sox6-WT and the NC mimic, Sox6-MUT and the NC mimic, and Sox6-MUT and the miRNA-499-5p mimic (Fig. 3C, D). These results confirmed that Sox6 is the direct target of miRNA-499-5p, which is consistent with previous studies (Li et al. 2013; Wang et al. 2017).

**Table 1:** Target genes found in both sequencing results and predicted results

Targeted gene	log_2_(FoldChange)	stat	*P*-value	*P* adj
Chdh	1.75	4.80	< 0.001	< 0.001
Chd9	1.18	7.61	< 0.001	< 0.001
Mpp7	1.02	3.79	< 0.001	< 0.01
Sox6	0.98	5.41	< 0.001	< 0.001
CTNNAL1	0.63	4.56	< 0.001	< 0.001
Mgme1	0.61	4.14	< 0.001	< 0.01
Cpt1a	0.53	3.01	< 0.01	< 0.05
Wdhd1	0.51	3.38	< 0.001	< 0.05
Mycbp	0.49	3.09	< 0.01	< 0.05
Ptpdc1	0.45	3.27	< 0.01	< 0.05
Bnc1	0.43	3.98	< 0.001	< 0.01
Tmem106b	0.34	3.23	< 0.01	< 0.05
Adss	0.33	3.63	< 0.001	< 0.01
Atad2	0.33	3.34	< 0.001	< 0.05
Nup155	0.31	3.76	< 0.001	< 0.01

*stat* standard error; *P adj* adjusted P-value

To evaluate the effect of Sox6-mediated propranolol on EMT in 4T1 cells, we employed two different sequence Sox6 siRNAs to knockdown the expression of Sox6 mRNA, of which Sox6 siRNA1 exhibited significant effects (Fig. 3E). The wound healing, cell migration, and invasion assay results showed that compared to 4T1 cells transfected with NC siRNA, the migration and invasion rate of cells transfected with Sox6 siRNA increased significantly (Fig. 3F–K). Western blot analysis indicated revealed that the expression of Sox6 and E-cadherin in 4T1 cells transfected with Sox6 siRNA decreased, while vimentin increased, when compared with cells transfected with NC siRNA. The downstream Akt signaling pathway, Wnt/β-catenin pathway, and Twist1 of Sox6 are considered to be closely related to EMT. The relevant Western blot results demonstrated that the expression of Wnt2, β-catenin, c-Myc, and p-GSK3β ser9 in 4T1 cells transfected with Sox6 siRNA was higher, GSK3β expression was lower, and t-Akt expression did not differ significantly compared to the NC group (Fig. 3L, M). These findings suggest that Sox6 knockdown leads to the activation of the downstream EMT-related Akt pathway, Wnt/β-catenin pathway, and Twist1. These results indicate that Sox6 is involved in the regulation of EMT, migration, and invasion of 4T1 cells.

**Fig. 4:**
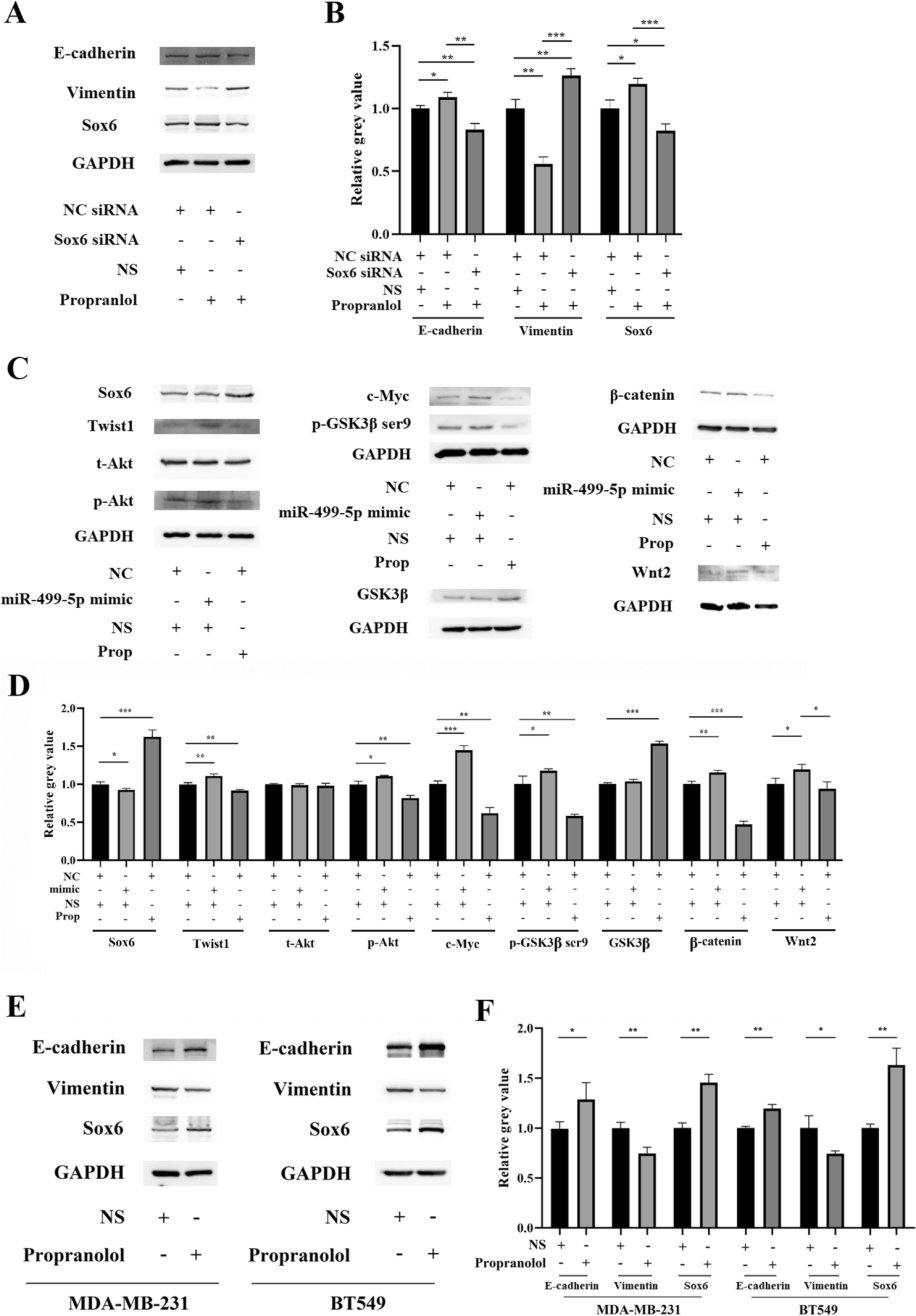
Propranolol inhibition of EMT via miR-499-5p is mediated by Sox6. **A**, **B** Western blot analysis of Sox6, E-cadherin, vimentin, and GAPDH protein in 4T1 cells transfected with NC or Sox6 siRNA and treated with 10 μM propranolol or NS for 72 h, and corresponding histogram normalized to GAPDH. **C**, **D** Western blot analysis of Sox6, EMT-related and Sox6 downstream-related proteins, and GAPDH in 4T1 cells transfected with NC or miR-499-5p mimic and treated with 10 μM propranolol or NS for 72 h, with corresponding histogram normalized to GAPDH. **E**, **F** Western blot analysis of Sox6, E-cadherin, vimentin, and GAPDH protein in MDA-MB-231 and BT549 cells treated with 10 μM propranolol or NS, with corresponding histogram normalized to GAPDH. *, *P* < 0.05; **, *P* < 0.01; ***, *P* < 0.001

To investigate whether the inhibitory effect of propranolol on EMT in 4T1 cells is primarily mediated by Sox6, we conducted simultaneous treatment of propranolol and Sox6 siRNA. Western blot analysis revealed that in comparison to the control group (NC siRNA and physiological saline treatment), the propranolol with NC siRNA group exhibited increased expression of E-cadherin and Sox6, while vimentin expression was decreased. In contrast, the propranolol with Sox6 siRNA group showed the opposite trend (Fig. 4A, B). These findings suggest that the inhibitory effect of propranolol on EMT in 4T1 cells is nullified when Sox6 mRNA is disrupted, further indicating the inhibitory effect of propranolol on EMT is primarily mediated by Sox6.

To investigate the effects of propranolol on Sox6 expression and its downstream EMT-related signaling pathways in 4T1 cells, we examined the changes in Sox6 and downstream protein expression using Western blot analysis. The results demonstrated that compared to the control group (NC mimic and physiological saline treatment), propranolol with NC mimic treatment upregulated the expression of Sox6 and GSK3β, while it downregulated the expression of p-Akt, Twist1, β-catenin, c-Myc, and p-GSK3β ser9 in 4T1 cells. In contrast, miR-499-5p mimic and physiological saline treatment exhibited opposite effects. Notably, there were no significant differences in t-Akt expression among the groups (Fig. 4C, D). These results suggest that propranolol can upregulate Sox6 and downregulate downstream EMT-related pathways/factors (Akt, Twist1, Wnt/β-catenin), while overexpression of miRNA-499-5p can downregulate Sox6 and upregulate these pathways/factors.

We further investigated whether the EMT inhibitory effects of propranolol are also present in other breast cancer cell lines by treating MDA-MB-231 and BT-549 cell lines with propranolol. Western blot analysis showed that propranolol also upregulated E-cadherin and Sox6 expression and downregulated vimentin expression in both cell lines (Fig. 4C, D). These results suggest that propranolol's EMT inhibitory effects, mediated by miR-499-5p and Sox6, may be present in various breast cancer cell lines.

Taken together, our results indicate that propranolol may downregulate miR-499-5p expression in 4T1 cells, leading to increased Sox6 expression and inhibition of Sox6 downstream EMT-related pathways (Akt, Twist1, Wnt/β-catenin), thereby inhibiting EMT and the occurrence of metastasis in 4T1 cells. Furthermore, this EMT inhibitory effect of propranolol may be present in various breast cancer cell lines (Table 1).

## Discussion

Propranolol is a well-established drug used primarily in the treatment of cardiovascular diseases (Friedman et al. 1986; Prichard and Gillam 1969), as well as infantile hemangiomas (Leaute-Labreze et al. 2008), hyperthyroidism (Murchison et al. 1979), and psychiatric disorders (Waal 1967). In recent years, there has been notable progress in research on propranolol in the field of cancer, with studies demonstrating its antitumor effects on neuroblastoma (Wolter et al. 2014) and pancreatic cancer (Partecke et al. 2016). It has been identified to reduce the expression of mesenchymal genes (Hiller et al. 2020), inhibit cell migration and invasion (Rico et al. 2017), and prevent metastasis in breast cancer (Choy et al. 2016; Rico et al. 2017). Our study validates and extends these findings, illustrating that propranolol effectively restrains EMT in breast cancer cells, leading to a reduction in lung metastasis.

EMT is a process where cells reduce or lose intercellular adhesion, undergoing depolarization and transforming into cells with mesenchymal characteristics (Gupta and Massagué 2006). While EMT is closely related to cell differentiation, embryonic development, and tissue repair, it also plays a pivotal role in tumor metastasis (Nieto et al. 2016). When tumor cells undergo EMT, there is a significant alteration in intercellular adhesion, invasion, and migration abilities, establishing the fundamental conditions for distant metastasis of tumors (Singh and Settleman 2010). E-cadherin and vimentin are crucial proteins that indicate the occurrence of EMT. E-cadherin is an essential component of the cytoskeleton and intercellular adhesion (Canel et al. 2013), while vimentin is a specific protein expressed by mesenchymal cells, indicating the initiation of EMT and the heightened invasive state of tumor cells (McInroy and Maatta 2007). In our study, propranolol downregulated vimentin and upregulated E-cadherin expression in breast cancer cells. Additionally, it effectively inhibited cell migration and invasion, aligning with previous findings (Shaashua et al. 2017).

Previous studies have identified that several signaling pathways, factors, and the extracellular environment are associated with the regulation of EMT(Lamouille et al. 2014). In our previous studies, we found that propranolol could reverse norepinephrine-induced tumor cell EMT through the modulation of β-AR/TGF-β1/HIF-1α/Snail and β-AR/TGF-β1/p-Smad3/Snail pathways (Zhang et al. 2016). Additionally, independent studies have highlighted propranolol's capability to block the activation of the β-AR/IL-6/Stat3/Snail pathway, leading to the reversal of EMT induced by isoproterenol (Liu et al. 2018). However, the role of miRNAs in the regulation of tumor cell EMT by propranolol has not been explored. Our study investigates this relationship by identifying miR-499-5p as a potential target. Previous studies have shown the effects of miR-499-5p on tumors, particularly in the context of EMT. A study reported significant upregulation of miRNA-499-5p in the metastatic cell line SW620 compared to the primary colon cancer cell line SW480 (Liu et al. 2011). Furthermore, it has been proven that miR-499-5p can promote the invasion and EMT in lung and colon cancer cells (He et al. 2019; Liu et al. 2011). Inhibition of miRNA-499-5p can upregulate the tumor suppressor factor CYLD, leading to the promotion of tumor cell apoptosis, inhibition of proliferation, and enhancement of E-cadherin expression (Long and Pi 2018). Our study indicated that the overexpression of miR-499-5p induces EMT of 4T1 cells, highlighting miR-499-5p as one of the paths through which propranolol exerts its inhibitory effect on EMT.

Sox6 is a member of the SoxD subfamily and is involved in organ development and various physiological processes (Bowles et al. 2000). Previous studies have suggested the potential involvement of Sox6 in the EMT of numerous tumor cells, engaging various downstream pathways/factors, including Twist1 (Wang et al. 2018), the Akt pathway (Jiang et al. 2018), and the Wnt/β-catenin pathway (Iguchi et al. 2007; Dong et al. 2018). Our study is in line with these findings, as we demonstrated that Sox6 knockdown induces EMT in 4T1 cells and activates downstream EMT-related pathways. Subsequent luciferase reporter assays confirmed that Sox6 is a direct target of miR-499-5p. We further revealed that Sox6 expression was increased with propranolol treatment and decreased with miR-499-5p overexpression. This modulation affected the EMT-related pathways downstream associated with Sox6, leading to the downregulation or upregulation of EMT levels. However, propranolol was unable to reverse the EMT upregulation caused by either miR-499-5p overexpression or knockdown of Sox6.

In summary, we have, for the first time, identified the role of miR-499-5p and its target Sox6 in the regulation of EMT and metastasis of 4T1 cells by propranolol. This mechanism may have broader implications for a variety of breast cancer cells. Subsequent studies are needed to further explore whether a similar mechanism and pathway apply to propranolol in human contexts.

## Methods

### Cell culture

The cell lines used in this study were all obtained from the American Type Culture Collection (ATCC) (Manassas, VA, USA). 4T1 and BT-549 cells were cultured in RPMI-1640 medium (Gibco, Carlsbad, CA, USA), MDA-MB-231 cells were cultured in Leibovitz's L-15 medium (Gibco, Carlsbad, CA, USA), and HEK-293 T cells were cultured in Dulbecco's modified Eagle's medium (Gibco, Carlsbad, CA, USA). All culture media were supplemented with 10% FBS. All cells were incubated in a sterile culture incubator at 37 °C, 95% saturated humidity, and 5% CO2, and the culture media were changed every 2 days.

### Mice model

1 × 10^5^ 4T1 cells were suspended in 100 μl of physiological saline and injected subcutaneously into the right mammary gland of each 6-week-old female BALB/c mouse (Beijing Huafukang Bioscience, Beijing, China). 7 days later, the mice were randomly divided into control and propranolol groups of 5 mice each. The mice in the propranolol group were given an intraperitoneal injection of 3 mg/kg of propranolol solution daily, while those in the control group were given an equal volume of sterile physiological saline (Liu et al. 2015) for 28 consecutive days. After executing the mice, the lungs were removed and washed in physiological saline, and the number of lung nodules on the surface of the lungs was counted. All animal experiments were conducted in accordance with the guidelines of the Animal Care and Use Committee of West China Hospital, Sichuan University.

### Sequencing

Second-generation sequencing (SGS) was performed using the Illumina HiSeq 2000 platform. miRNAs and mRNAs extracted from tumor tissues in animal models were used for sequencing. Sequencing data were analyzed using miRDeep2 (2.0.0.8) after removing adapter sequences, reads less than 15 bases in length, etc., and miRNA and mRNA data were mapped to miRBASE (release 22.1, allowing zero mismatches), subsequently, quantified and analyzed for differences in expression of miRNAs, mRNAs in the control, and propranolol groups using the DEseq2 R package. The threshold of significance was *P* < 0.05 and |log2 (FoldChange)|> 2.

### miRNA, siRNA, and plasmid transfections

4T1 and HEK-293 T cells were seeded at a density of 5 × 10^4^ cells per well in a 12-well plate and incubated for 24 h. The cells were transfected with miRNA mimics, siRNA(GeneCopoeia, Guangzhou, China) or reporter gene (has been cloned into psi-CHECK™-2 vector) plasmid (Tsingke Bio Co., Beijing, China) using Lipofectamine RNAiMAX Transfection Reagent (Invitrogen, Carlsbad, California, USA) or Lipofectamine 3000 reagent (Invitrogen, Carlsbad, California, USA), respectively, and the cells were collected after 6 h of transfection for the next step.

### RT-PCR

The miRNA or mRNA was extracted and reverse transcribed into cDNA using miRcute Plus miRNA First-Strand cDNA Kit (contains a proprietary universal RT primer. Catalog: KR211-01, TIANGEN, Beijing, China) or PrimeScript RT reagent Kit (Takara, Shiga, Japan), followed by expression analysis using LightCycler 96 System (Roche, Basel, Switzerland) (U6 or GAPDH was used as internal controls for miRNA and mRNA, respectively). miRNA or mRNA was analyzed using miRcute Plus miRNA qPCR Kit (Contains a proprietary universal reverse primer. Catalog: FP411-01, TIANGEN, Beijing, China) or TB Green® Premix Ex Taq™ II (Takara, Shiga, Japan). Primer sequences were followed (5′ to 3′): mmu-miR-206-3p forward: GCAGTGGAATGTAAGGAAGT; mmu-miR-499-5p forward: GGGGTTAAGACTT-GCAGTG; mmu-miR-1a-1-5p forward: ACAUACUUCUUUAUAUGCCCAUA; mmu-miR-133a-5p forward: GCTGGTAAAATGGAACCAAAT; mmu-miR-133a-3p forward: TTTGGTCCCCTTCAACCAGCTG; mmu-miR-1a-3p forward: ACGATGG-AATGTAAAGAAGT; mmu-miR-133b-3p forward: TTTGGTCCCCTTCAACC-AGCTA; mmu-miR-124-3p forward: TAAGGCACGCGGTGAATGCC; mmu-miR-708-5p forward: AAGGAGCTTACAATCTAGCTGGG; mmu-miR-708-3p forward: CAACTAGACTGTGAGCTTCTAG; u6 forward: CTCGCTTCGGCAGCACA; Sox6 forward: CCCCTCTGAACATGGTGGTGGC, reverse: TGAGACTGCCCCTGC-CGAGT; and GAPDH forward: AGCAGTCCCGTACACTGGCAAAC, reverse: TCTG-TGGTGATGTAAATGTCCTCT. miRNA primers were provided by GeneCopoeia Inc. (Guangzhou, China) and mRNA primers were provided by GenePharma Co (Shanghai, China).

### Western blots

Total protein was extracted from tumor tissue or cells using RIPA lysis buffer (Beyotime, Shanghai, China), and the protein concentration was determined using a BCA protein assay kit (Solarbio, Beijing, China). Equal amounts of sample were then subjected to electrophoresis for an appropriate time and transferred to a polyvinylidene fluoride membrane (Bio-Rad, California, USA). The membrane was blocked with non-specific antibodies and probed with primary antibodies, including rabbit anti-mouse antibodies against CTNNAL1, c-Myc, GSK3β, Sox6, p-Akt, t-Akt (ZenBio, Chengdu, China), E-cadherin (Proteintech, Wuhan, China), Twist1, Wnt2, β-catenin (Huabio, HangZhou, China), and vimentin (Bioss, Beijing, China), as well as a mouse anti-mouse antibody against GAPDH (ZenBio, Chengdu, China). The membrane was then incubated with HRP-labeled goat anti-rabbit (ZenBio, Chengdu, China) and goat anti-mouse (ZenBio, Chengdu, China) secondary antibodies at room temperature. Finally, the membrane was treated with Immobilon Western HRP Substrate (Millipore, Bedford, MA, USA) and detected on an iBright CL1000 Imaging System (Invitrogen, Carlsbad, California, USA). Image J software was used to analyze the relative intensity of the bands.

### Bioinformatics

To investigate whether miRNA may be involved in the EMT inhibitory effect of propranolol, we performed bioinformatics analysis of the differential expression of miRNA and mRNA between the control and intervention groups in the SGS results.

Enrichment analysis: Enrichment analysis categorizes genes based on known annotation information to summarize their commonalities in certain aspects. miRNAs that showed significant expression differences (|Log2(FoldChange)|> 2) between the propranolol group and the control group were used for enrichment analysis. The analysis was performed using the DIANA online tool (http://diana.imis.athena-innovation.gr/), which includes three miRNA target gene prediction databases (miRTarBase, microT-CDS, and TargetScan). The analysis was based on the annotation of the Kyoto Encyclopedia of Genes and Genomes (KEGG) and Gene Ontology (GO) databases. The KEGG database provides annotation for genes in terms of chemical and systemic functions, as well as relevant pathways, while the GO database annotates genes in terms of molecular biological functions, biological processes, and cellular components. The results of miRNA enrichment analysis are presented in the form of a heatmap, where each row represents a miRNA and each column represents a relevant domain or pathway. The color of the squares becomes redder as the *P*-value decreases. Enrichment analysis of mRNAs showing significant expression differences (*p* < 0.05) was performed using the Omicshare online tool (https://www.omicshare.com/tools/), also based on KEGG and GO annotations. The results are presented in the form of a bubble plot, where the X-axis represents the proportion of differentially expressed genes in that pathway relative to the total number of differentially expressed genes. The color change represents the corresponding P-value, ranging from the smallest blue to the largest red. The size of the dots represents the number of genes included in the analysis, with larger dots indicating a larger number of genes.

Prediction of miRNA target genes and comparison with sequencing results: TargetScan 7.2 (http://www.targetscan.org/) and DIANA (http://diana.imis.athena-innovation.gr/) were used as miRNA target gene prediction tools to predict potential target genes and corresponding binding sites of miRNA-499-5p. mRNA showing upregulated expression in the sequencing results and identified as potential targets in the prediction results was further compared in the literature.

### Immunohistochemistry

Tumor tissues were fixed using 4% paraformaldehyde for 24 h at room temperature. Tumor tissues were embedded in paraffin and then sectioned. The sections were incubated at 65 °C for 30 min and then placed successively in xylene (15 min), 100% ethanol (5 min), 95% ethanol (2 min), 90% ethanol (2 min), 80% ethanol (2 min), and then in citric acid buffer heated to 97 °C for 40 min, followed by immersion in 3% H2O2 for 30 min. Non-specific antigens were blocked using goat serum, followed by incubation (37°C, 2 h) with primary antibodies: rabbit anti-mouse E-cadherin from Proteintech (Wuhan, China) and chicken anti-mouse vimentin from Abcam (Cambridge, UK). An HRP-labeled secondary antibodies (Abcam, Cambridge, UK) was then used for incubation at 37 °C for 30 min. Hematoxylin (Bioss, Beijing, China) was used to re-stain the sections and to seal the sections. An electron microscope (Zeiss, Oberkochen, Germany) was used to visualize and photograph the sections.

### Immunofluorescence

4T1 cells were inoculated into a 24-well culture plate with pre-placed cell slides. The cells were treated with 4% paraformaldehyde for 15 min and then placed in 0.2% Triton X-100 at 4 °C for 5 min. Non-specific antibodies were blocked with goat serum containing 0.3% Triton X-100 (room temperature, 1 h). The mixed primary antibodies, including E-cadherin (CST, Boston, Massachusetts, USA) and vimentin (Abcam, Cambridge, UK), were placed over the slides and incubated for 12 h at 4 °C in a light-proof environment. Alexa Fluor 594 and Alexa Fluor 488 were coupled to the above two primary antibodies and the nuclei were stained with DAPI staining solution (Invitrogen, Camarillo, CA, USA). The slides were sealed and observed under a fluorescence confocal microscope (Zeiss, Oberkochen, Germany) and photographed.

### Dual-luciferase reporter gene assay

pEZX-FR02-WT-Sox6 (Sox6-WT) containing the 3′-UTR region and pEZX-FR02-MUT-Sox6 (Sox6-MUT) with mutated binding site with miR-499-5p (Tsingke Bio Co., Beijing, China) were constructed and co-transfected with the two combinations of negative control mimic and miR-499-5p mimic into HEK-293 T cells, and the reporter plasmid was constructed containing only three 7mer-m8 sites, consistent with a previous study(Li et al. 2013). Cells were processed using the dual-luciferase reporter gene assay kit (Beyotime, Shanghai, China) and fluorescence intensity was detected on a SpectraMAX i3x Platform (Molecular Devices, California, USA).

### Wound healing assay

The 4T1 cells were inoculated in 6-well plate culture. When the cells covered the bottom surface of the plate, a sterile 200-μL pipette tip was used to gently and evenly draw lines of approximately the same width on the bottom surface, followed by washing the detached cells and debris, and the culture medium was replaced with RPMI-1640 medium without FBS (Rahimi et al. 2018). The cells were photographed at hour 0, hour 12, and hour 24 using an OLYMPUS inverted microscope (Tokyo, Japan) at pre-selected sites. The width between cells was analyzed using ImageJ software.

### Transwell cell migration and invasion assays

Pre-culture 4T1 cells with serum-free medium for 4 h. A 100 μL of 4T1 cell resuspension was added to the transwell insert (8.0 µM, Corning Falcon, NY, USA) with 1.5 × 10^5^ cells and incubated at 37 °C for 24 h. Invasion experiments were performed by adding extracellular matrix materials (Matrigel, BD Biocoat, Massachusetts, USA) to the transwell insert in advance. The transwell insert was removed and placed in 4% paraformaldehyde for 15 min to remove cells from the supramembrane surface of the transwell insertion. The cells on the membrane were stained with 0.1% crystalline violet dye (Beyotime, Shanghai, China) and then placed under an OLYMPUS inverted microscope (Tokyo, Japan) for observation and photography.

### Statistical analysis

Statistical Product Service Solutions (v17.0, SPSS, Chicago, IL, USA) was used for statistical analysis of the experimental data. Statistical significance was performed using *t*-test, chi-square test, or rank sum test (based on chi-square). *p* < 0.05 indicates that the differences are statistically significant.

## Data Availability

The datasets generated during and/or analyzed during the current study are available from the corresponding author on reasonable request.
